# Complete Genome Sequence of Infectious Bronchitis Virus Strain JP/KH/64, Isolated in Japan

**DOI:** 10.1128/MRA.00665-21

**Published:** 2021-10-07

**Authors:** Masaji Mase, Kanae Hiramatsu, Satoko Watanabe, Hiroshi Iseki

**Affiliations:** a National Institute of Animal Health, National Agriculture and Food Research Organization, Tsukuba, Ibaraki, Japan; b Oita Livestock Hygiene Service Center of Oita Prefecture, Oita, Oita, Japan; c United Graduate School of Veterinary Sciences, Gifu University, Gifu, Gifu, Japan; Queens College CUNY

## Abstract

Here, we report the complete genome sequence of infectious bronchitis virus (IBV) strain JP/KH/64, which is the reference strain for the JP-I genotype in Japan. This information should be useful for an in-depth understanding of the evolution of the JP-I genotype.

## ANNOUNCEMENT

Avian infectious bronchitis virus (IBV) belongs to the genus *Gammacoronavirus* in the family *Coronaviridae* of the order *Nidovirales* (1). This highly contagious pathogen is transmitted through the respiratory tract and causes infectious bronchitis in chickens, leading to serious economic consequences worldwide (1). IBV has three major virus-encoded structural proteins, i.e., the spike (S) glycoprotein, the membrane (M) protein, and the nucleocapsid (N) protein. Among these proteins, the S1 glycoprotein is associated with virus attachment and is a major target of neutralizing antibodies in chickens (2–4).

In Japan, seven genotypes of IBV (JP-I, JP-II, JP-III, JP-IV, Mass, Gray, and 4/91) have been confirmed, based on the partial nucleotide sequence of the S1 gene (5, 6). Analysis of genes other than S1 revealed that various recombinant viruses are also prevalent in Japan (6). A complete genome analysis of the IBV is important for understanding the epidemiology of recombinant viruses. To understand the epidemiology of variant IBV in Japan, the complete nucleotide sequence of the major genotype JP-I strain was determined.

The JP/KH/64 strain was isolated from chickens with respiratory symptoms in 1964 in Japan and was first confirmed as the JP-I genotype in Japan (5). The virus was grown in primary chicken kidney cell cultures prepared from 4- to 10-week-old chicks, and the infected culture fluids were harvested. Viral RNA was extracted from infected culture fluids using a QIAamp viral RNA minikit (Qiagen, Hilden, Germany), and random hexamer primers were used for cDNA synthesis (6). Specific primers for genome sequencing using the Sanger method were designed based on previous studies (7, 8) and on the sequences obtained from amplicons (Table 1). Both the 3′ and 5′ termini of the genome were determined using the rapid amplification of cDNA ends (RACE) technique.

**TABLE 1:** Primers used to amplify the complete genome sequence of JP/KH/64

Nucleotide positions^*a*^	Forward primer	Reverse primer
19–1645	TATATATCTATTGCACTAGCC	AGTCAGACAGACAACACGCT
1437–3080	CACAAGTTGTTGTTCGTGGC	GAGCGTCCTTATGAACTATAC
2893–3851	AGATGCTGAGGAGTGTGATAC	AGTTACTGTCTCCATGGCGTG
3742–5610	GGACTATGTTAAGAAACATGG	CCATCTCCTACTTTATCGGC
5438–7344	GTTGATGGTGTAACTATGGGC	GTACCACTAACGATACCACC
7223–9224	TCAGCGACTGTCAAGTCAGG	TACCACCATAAGAGCAAGC
9017–9307	CAATGGAGTGATGTACTTAATC	AACTGAGCCACAAGCTCCTGC
9176–10950	GAAAGCAAATTGTGGTGATAG	GAGGGAATGTGTGAAAACTC
10816–12586	GGACAATTTGTTGGGTATGC	GTTCATAATTACTAGGAGTGG
12412–14028	CCTGATGTTGTAGAGCGAGCC	AAGAATGGACACACCTGCCAC
13150–13955	CCCTCCTCAAGTATGATTAT	ATAGTGGGCAGGACATTCTT
13929–14875	TACAAAGAAGAATGTCCTGCC	CATCTACAAAAACACATGCACC
14813–15647	AGCCTAAATACTTGCCATATCCAG	TTACCTGGCTCCCATGATAGAATC
15520–16868	TCATTAAGACGCTTTGCTG	ATGACTACAAGTATACCACGC
16738–19004	GTAGACTCTTCACAAGGTTC	TAAACATACAGATTCGCTCC
17318–18797	TAGGCAATAATTTTGAGCCTG	TTAACAGTATTACGATATAATGG
18402–20306	GGCTTCTTATAATGCAGCTG	AAATTCACAACTGGTGTTGC
20071–22297	CAAGATTGTGCATGGTGGAC	TATGTTATCACAAACAGGACC
20959–21902	CTTTAAAGCAGGCGGACCTATAAC	TACCACCTGATACTACAAACTGCTG
21275–22976	ACACAAACAGCTCAGAGTGG	CTTGAATTGCATCAAGTTGC
22801–24896	CATTGGTCATATGCAGGAAGG	GGAGTATTGAACCTACGGCATTA
23757–24991	GTTGTTGTTGTGGATGCTTTGG	CTGACCTTCACAATAAAGAAC
24764–25867	GCAGCGATAATACTTACAGT	TCTGCTTGTCCTGCTTTGT
25089–26426	GTGACCGAAGCGGAAATAA	TCAGAGGAATGAAGTCCCAAC
26264–27672	GGTGATTCTCAAGATGGTAT	GCTCTAACTCTATACTAGCCT
1–157	AUAP^*b*^	AAAACCTGACAGGTGGCCAG
1–202	AAP^*b*^	GGCAAAACAAGCAGTGATAC
27448–27672	TTAGTAGCCTGGAAACGAACGGTAG	UAP^*b*^

a Primer locations are listed according to the JP/KH/64 strain.

b The abridged universal amplification primer (AUAP), 5′-RACE abridged anchor primer (AAP), and universal amplification primer (UAP) are included in the commercial RACE kit (Invitrogen).

The sequenced fragments were assembled using ATGC-Mac v.5 (Software Development, Japan). All tools were run with default parameters unless otherwise specified. The length of the complete genome of JP/KH/64 was 27,672 nucleotides (nt), with a G+C content of 38.20%, excluding the poly(A) tail. The open reading frames (ORFs) were identified using Genetyx-Mac v.18 (Software Development) and were confirmed by alignment with previously published IBV genome sequences. The genome has the typical genetic structure of all IBV strains (9) and contains 13 ORFs (5′-1a-1ab-S-3a-3b-E-M-4b-4c-5a-5b-N-6b-3′), 11,859 nt, 19,893 nt, 3,510 nt, 174 nt, 195 nt, 324 nt, 672 nt, 285 nt,171 nt, 198 nt, 249 nt, 1,230 nt, and 222 nt in length, respectively.

**FIG 1:**
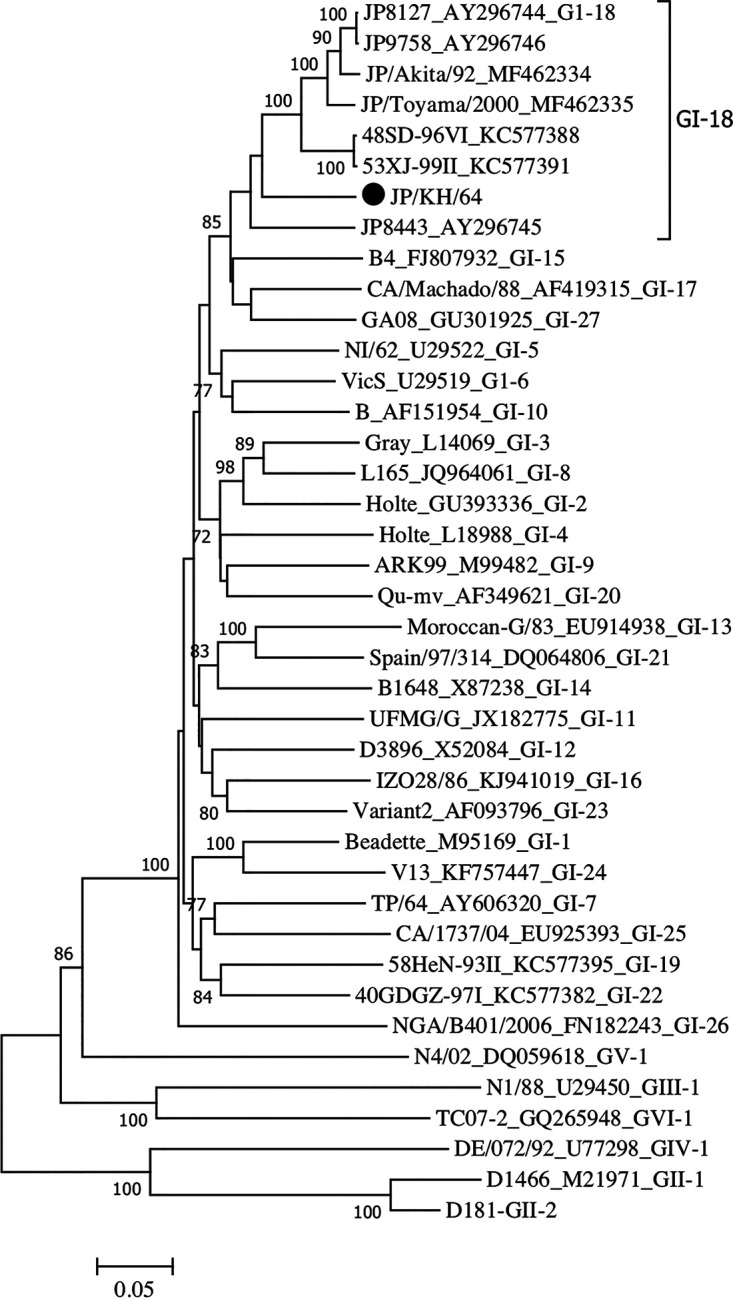
Phylogenetic tree based on the complete S1 glycoprotein gene of IBV. Nucleotides 20368 to 21978 (1,632 bases) of the S1 gene of IBV Beaudette (GI-1) (GenBank accession number NC_001451) were subjected to phylogenetic analysis. The phylogenetic tree was generated using the neighbor-joining method in MEGA 7 (14) with 1,000 bootstrap replications. All tools were run with default parameters unless otherwise specified. Horizontal distances are proportional to the minimum number of nucleotide differences required to join nodes and sequences. The genotypes of IBV were defined by Valastro et al. (10). The JP/KH/64 strain is indicated with a black circle.

IBV is classified into six main genotypes (GI to GVI), comprising 32 viral lineages (lineages 1 to 32), based on the complete nucleotide sequences of the S1 glycoprotein and its typical geographical distribution (10). Phylogenetic analysis based on the complete coding sequence of the S1 gene reconfirmed that JP/KH/64 is a member of the GI-18 lineage, clustering in one group with the lineage prototype strain JP8127 (GenBank accession number AY296744) (87.6% nucleotide identity), which was isolated in Japan (11) (Fig. 1). Because the JP-I genotype has been reported in China, Taiwan, and Japan (12, 13), this information about the JP/KH/64 strain might be important for the understanding of IBV evolution in Eastern Asian countries.

### Data availability.

The genome sequence of IBV strain JP/KH/64 was deposited in GenBank under accession number LC634083.
